# Oral Iron Supplementation in Pregnancy: Current Recommendations and Evidence-Based Medicine

**DOI:** 10.1055/s-0041-1736144

**Published:** 2021-11-16

**Authors:** Ana Filipa Moreira Duarte, Ana Catarina Simões Viana Carneiro, Ana Teresa Barbosa Maciel Meira Peixoto, Daniela Filipa Pereira Montenegro, Débora Sofia Carvalho Campos, Ana Patrícia Ribeiro Alves, Ana Rita Mota Magalhães Costa, Andreia Patrícia Machado Fino

**Affiliations:** 1 USF Alcaides de Faria - ACES Barcelos-Esposende - ARS Norte; 2 USF Santo António- ACES Barcelos-Esposende - ARS Norte

**Keywords:** iron, dietary supplements, pregnancy, anemia, prenatal care, ferro, suplementação nutricional, gravidez, anemia, cuidado prey-natal

## Abstract

**Objective** To review the evidence about universal iron supplementation in pregnancy to prevent maternal anemia.

**Methods** Bibliographic research of randomized and controlled clinical trials, meta-analyses, systematic reviews, and clinical guidelines, published between August 2009 and August 2019, using the MeSH terms: *iron;* *therapeutic use* ; *pregnancy* ; *anemia* , *prevention* *and* *control* .

**Results** We included six clinical guidelines, three meta-analyses and one randomized controlled clinical trial.

**Discussion** Most articles point to the improvement of hematological parameters and reduction of maternal anemia risk, with supplementary iron. However, they do not correlate this improvement in pregnant women without previous anemia with the eventual improvement of clinical parameters.

**Conclusion** Universal iron supplementation in pregnancy is controversial, so we attribute a SORT C recommendation strength.

## Resumo

**Objetivo** Rever a evidência sobre a necessidade de suplementação universal de ferro na gravidez para prevenção de anemia materna.

**Métodos** Pesquisa bibliográfica de ensaios clínicos aleatorizados e controlados, metanálises, revisões sistemáticas e normas de orientação clínica, publicados entre agosto de 2009 e agosto de 2019, utilizando os termos MeSH: *iron, terapêuticas use; pregnancy; anemia, preventivos and control.*

**Resultados** Incluímos seis normas de orientação clínica, três metanálises e um ensaio clínico randomizado e controlado.

**Discussão** A maioria dos artigos aponta para a melhoria dos parâmetros hematológicos e redução do risco de anemia materna por meio da suplementação com ferro. Todavia, eles não correlacionam a melhoria destes parâmetros em grávidas sem anemia prévia com a eventual melhoria de parâmetros clínicos.

**Conclusões** A suplementação universal com ferro na gravidez é controversa, pelo que atribuímos uma força de recomendação SORT C.

## Introduction

Gestational anemia is the most common health problem in pregnancy. Its prevalence varies with geographic region, and, according to data form the World Health Organization (WHO), the global prevalence is 38.2%, and ∼ 26% in Europe. In developing countries, the prevalence is higher, reaching 48.7% in Southeast Asia and 46.3% in Africa. 1 On the other hand, the estimated prevalence of iron deficit in pregnant women in the USA is 18.6%, and 16.2% of these have anemia. 2 Taking into account the World Development Indicators of the Global Health Observatory, The World Bank, the prevalence of gestational anemia in 2016 in Brazil was 37.3%, and 25% in Portugal. 3 In Portugal, the EMPIRE study described a prevalence of anemia of 54.2% in pregnant women, with regional variability, with ferropenia being the most frequent cause. 4 5

Ferropenic anemia is associated with an increase in maternal morbidity, such as greater severity or susceptibility to infection, increased risk of peripartum transfusion, pre-eclampsia, premature detachment of normally inserted placenta, and may culminate in maternal death. 5 6 Maternal anemia may also increase the risk of postpartum hemorrhage. 7

Currently, there is an inconsistency between the recommendations of the clinical guidelines (CG) of the Portuguese Directorate-General of Health (Direção-Geral da Saúde [DGS, in the Portuguese acronym) 8 because the National Low-Risk Pregnancy Surveillance Program of recommends iron supplementation in all pregnant women from 14 weeks of gestation on, but the CG of the DGS on the approach, diagnosis and treatment of ferropenia suggests serum ferritin determination before starting iron at the first consultation and at 28 weeks. 9 Furthermore, universal supplementation with iron during pregnancy is not widely practiced by obstetricians.

The aim of the present review is to study the evidence on the need for oral iron supplementation in all pregnant women to prevent maternal anemia.

## Methods

We conducted a bibliographic survey of CGs, systematic reviews (SRs), meta-analyses (MAs) and randomized controlled clinical trials (RCTs) in August 2019 in the PubMed, National Guideline Clearinghouse, Guidelines Finder, Canadian Medical Association Practice Guidelines Infobase, The Cochrane Library and Evidence Based Medicine online, SciELO, and DGS databases, considering the articles published in the past 10 years in English and in Portuguese. We used the MeSH search terms *iron* ; *therapeutic use* ; *pregnancy* ; *anemia* ; and *prevention and control* , at PubMed and The Cochrane Library. We used the keywords *anemia* and *pregnancy* in the other databases, since these do not integrate search by MeSH terms. As inclusion criteria, we considered the studies whose target population consisted of asymptomatic pregnant women; that is, without established anemia, where we evaluated the prevention of maternal anemia as a result, through supplementation with oral iron, comparing it with nonsupplement. We excluded articles divergent from the objective of the work, repeated articles or included in selected MA/RS, nonrandomized clinical trials, classic reviews, articles not accessible online, articles whose intervention was in pregnant women with already known anemia or ferropenia, evaluations only in twin pregnancies, evaluations in pregnant women with major comorbidities, trials in which the iron intervention group was associated with another compound (for example, folic acid) and was not compared with the control group comprising the same compound, or where the intervention was supplementation with iron in nonoral formulations.

We determined the level of evidence and strength of recommendation using the American Family Physician Strength of Recommendation Taxonomy (SORT) scale. 10 This scale classifies articles according to 3 levels of evidence (level of evidence 1: studies with patient-oriented evidence of good quality; level of evidence 2: studies with patient-oriented evidence of limited quality; evidence level 3: studies with disease-oriented evidence) and 3 degrees of recommendation strength (recommendation strength A: patient-oriented evidence consistently; recommendation strength B: patient-oriented evidence of inconsistent or limited quality; recommendation strength C: disease-oriented and consensus-based evidence).

## Results

**Fig. 1:**
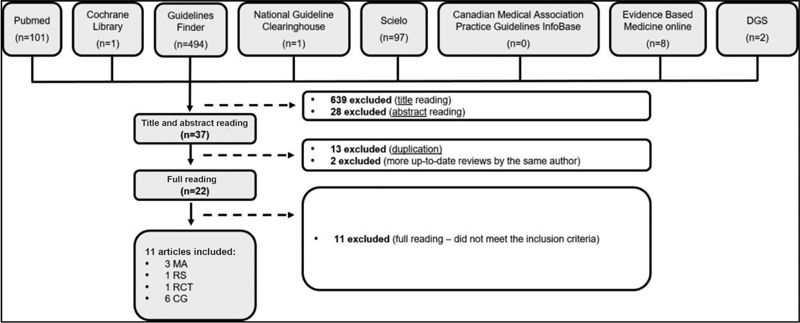
Illustrative scheme of article selection. CG – Clinical Guideline; DGS – Directorate-General of Health; MA - Meta-analysis; MA - Meta-analysis; RCT - Randomized and Controlled Clinical Trial; SR - Systematic Review.

In the initial research, we identified a total of 704 articles, of which we excluded 639 by the title, that is, because they did not fit the objective of the present review. Of the total of 65 articles, we excluded 28 by reading the abstract and 11 after full reading, because they did not meet the inclusion criteria. We also excluded duplicate 13 articles and 2 because there are more up-to-date reviews by the same author. Thus, we included 11 articles, of which 6 were CGs, 3 MAs, 1 SR and 1 original article, as shown in Fig. 1 .

The MA of Imdad et al, 11 published in 2012 ( Table 1 ), aimed to evaluate the impact of universal iron supplementation on maternal anemia and on perinatal results.

**Table 1:** Included articles

Reference/Type of study	Findings	LE
Imdad et al. (2012) 11 *MA*	• Daily supplementation of iron: ○ 69% reduction in the incidence of anemia at term in the intervention group compared with control (Relative Risk (RR) 0.31; 95% confidence Interval [CI]: 0.22–0.44]); ○ 66% reduction in ferropenic anemia at term (RR 0.44 [95% CI: 0.28–0.68]) compared with no intervention/placebo; • There was no statistically significant difference between intermittent administration of iron/iron-folic acid and daily administration, based on 3 studies. (RR 1.61 [95%CI: 0.82–3.14])	**1**
Haider et al. (2013) 12 *MA*	• Daily iron supplementation (effects in the 3 ^rd^ trimester and delivery): ○ increased the mean maternal Hb concentration by 4.59 g/L (95%CI: 3.72–5.46) compared with the control group; ○ significantly reduced the risk of anemia (RR 0.50; 95%CI: 0.42–0.59); ○ significantly reduced iron deficiency (RR 0.59, 95%CI: 0.46–0.79) and ferropenic anemia (RR 0.40, 95%CI: 0.26–0.60). • The effects on Hb concentration in the 2 ^nd^ trimester were not evaluated by a small number of trials.	**2**
Peña-Rosas et al. (2015) 13 *SR*	• Preventive iron supplementation reduces: ○ maternal anemia at term in 70% (RR 0.30; 95%CI: 0.19–0.46); ○ ferropenic anemia at term (RR 0.33; 95%CI: 0.16–0.69); ○ iron deficit at term by 57% (RR 0.43; 95%CI: 0.27–0.66); ○ Women who received added iron supplement increased risk of hemoconcentration at the end of pregnancy (average RR 3.07; 95%CI: 1.18–8.02) • The implementation of iron supplementation in all pregnant women can lead to heterogeneous results, since the risk of anemia is different between the various populations studied, as well as the level of adherence.	**2**
Abraha et al. (2019) 14 *MA*	• Any supplement containing iron reduced maternal anemia by 67% (RR 0.32; 95%CI: 0.20–0.49) with significant heterogeneity; ○ the subgroup analysis of studies using doses ≥ 200 mg/day (lower heterogeneity) obtained results consistent with the main analysis (RR 0.15; 95%CI: 0.08–0.28; participants = 360; studies = 5). • Iron-containing supplements reduced the incidence of ferropenic anemia, with no statistically significant difference (RR 0.33; 95%CI: 0.16–0.69); • The supplementation with iron resulted in a higher frequency of side effects compared with the control group (22 versus 18%), but with no statistical difference (RR 1.42; 95%CI: 0.91–2.21).	**2**
Parisi et al. (2017) 15 *RCT*	• **Levels of Hb:** ○ Concentration decreased in all groups; less pronounced decrease in liposomal iron groups (positive effect of interaction between time and groups – LI14 *p* < 0.03 and LI28 *p* < 0.001); ○ There were no statistically significant differences in Hb values between the control group and the ferrous iron group. • **Ferritin levels:** ○ statistically significant differences between ferritin concentration between liposomal iron and control groups (LI14 *p* < 0.02 and LI28 *p* < 0.001); • High incidence of iron deficit/iron anemia in the control group and with ferrous iron (30%).	**2**

Abbreviations: CI, Confidence Interval; Hb, Hemoglobin; LE, Level of Evidence; MA, Meta-analysis; RCT, Randomized and Controlled Clinical Trial; RR, Relative Risk; SR, Systematic Review.

A total of 14 of the 18 studies that evaluated the parameter “maternal anemia at term” considered the intervention to be oral iron alone. A subgroup analysis was performed comparing studies with iron and iron in combination with folic acid, demonstrating similar results. Thus, from the combined analysis of the 18 studies, they concluded that there was a significant reduction (69%) in the intervention group compared with the control group. There was no statistically significant difference between intermittent administration of iron/iron-folic acid and daily administration, based on 3 studies (data not shown). As a limitation of this MA, significant heterogeneity was identified, especially due to the prevalence of anemia in the various places of the included studies being different. The authors conclude that prophylactic iron supplementation during pregnancy has a significant benefit in reducing the incidence of maternal anemia, classifying the quality of the results as “moderate.”

The MA of Haider et al., 12 published in 2013 ( Table 1 ), aimed to summarize the evidence on the association of maternal anemia and iron supplementation in pregnancy with maternal hematological effects and adverse outcomes in pregnancy; it also evaluated the potential relationship between iron exposure (regarding dose, duration of use and hemoglobin [Hb] concentration) and maternal outcomes. A total of 48 randomized clinical trials ( *n* = 17,793) and 44 cohorts ( *n* = 1,851,682) were included. Regarding maternal Hb concentration, 36 trials evaluated this parameter in the 3 ^rd^ trimester and at the time of delivery and concluded that it was significantly higher in the iron supplemented group, with or without folic acid; heterogeneity was not found in this analysis. The effect of the intervention on the prevention of maternal anemia was evaluated in 19 trials. The use of iron, with or without folic, acid led to a 50% reduction in the risk of anemia in the 3 ^rd^ trimester or at delivery, with significant heterogeneity. The effects on Hb concentration in the 2 ^nd^ trimester were not evaluated because it was a small number of trials. The analysis also showed significant reductions in iron deficit in 8 trials and in iron deficiency anemia in 6 trials. Haider et al. 12 concluded that iron supplementation in women during pregnancy can be used as a preventive strategy to improve hematological status, advocating that prenatal anemia and iron deficiency were identified as preventable risk factors for developing disease.

Peña-Rosas et al. 13 conducted a systematic review published in the Cochrane Library in 2015 ( Table 1 ) whose objective was to evaluate the effect of iron supplementation in pregnant women as a public health intervention in antenatal care. A total of 44 studies were included ( *n* = 43,274 women), which compared the effects of daily iron supplementation versus no iron or placebo. The results regarding maternal outcomes showed that preventive iron supplementation reduced maternal anemia at term by 70% (relative risk [RR] 0.30; 95% confidence interval [CI]: 0.19–0.46; 14 trials, 2,199 women; low quality evidence), iron-deficiency anemia at term (RR 0.33; 95%CI: 0.16–0.69; 6 trials; 1,088 women), and iron deficiency at term by 57% (RR 0.43; 95%CI: 0.27–0.66; 7 trials; 1,256 women; low quality evidence). The authors concluded that iron supplementation reduces the risk of maternal anemia and iron deficiency during pregnancy. The implementation of iron supplementation in all pregnant women can lead to heterogeneous results depending on the risk of anemia in the population in question, as well as the level of adherence to the measure.

The MA of Abraha et al., 14 published in 2018 ( Table 1 ), aimed to evaluate and summarize the evidence of systematic reviews regarding oral iron administration to prevent critical pregnancy outcomes, to facilitate the formulation of health recommendations and policies. 14 Regarding the prevention of maternal anemia at term, the results showed that any supplement containing iron reduced maternal anemia by 67%, with significant heterogeneity; an analysis of the subgroup of studies using doses ≥ 200 mg/day was then performed, since they presented lower heterogeneity. In this analysis, account was taken of the fact that not all studies considered the control group to have the same micronutrients or other vitamins that were associated with iron in the intervention group. Thus, based on these studies with better quality, they concluded that the results are consistent with the main analysis. Iron supplements also reduced the incidence of ferropenic anemia by 67%, with no statistically significant difference. Iron supplementation resulted in a higher frequency of side effects compared with the control group (22 versus 18%), with no statistical difference. In conclusion, evidence of moderate quality was found to support the use of iron to prevent the incidence of anemia in pregnant women. This meta-analysis has limitations, especially regarding the variability of the type of iron supplementation – while most reviews considered the comparison of iron supplementation with placebo or non-treatment, there is a review whose objective was to evaluate the impact of supplementation with various micronutrients on pregnancy outcomes, in comparison with supplementation with iron alone or with folic acid, not fulfilling the objectives of our review, which may cause interpretation bias.

The RCT of Parisi et al., 15 published in 2017 ( Table 1 ), aimed to evaluate the effect of conventional ferrous iron and liposomal iron formulations, compared with a placebo control group, on maternal reserves during pregnancy. The exclusion criteria were: pregnant women with known pathology, use of chronic medication or any micronutrient supplementation in the first trimester of pregnancy, except for folic acid, extreme body mass index (BMI) (BMI < 18kg/m ^2^ or BMI > 30kg/m ^2^ ), serum Hb < 10.5 g/dl and/or ferritin < 15 mg/l in the selection phase, known fetal pathologies, and pregnancy complications. In addition, women with a vegetarian or vegan diet or any food restriction (allergies or food intolerance) were excluded. The total sample consisted of 80 pregnant women, randomized into 4 groups (supplementation into 3 groups and 1 control group with placebo, 20 women in each: group 1 with 30mg/ferrous iron day; group 2 with 14mg/day liposomal iron (LI14); group 3 with 28mg/day liposomal iron (LI28); group 4, control group, without supplementation). The authors concluded that iron supplementation during pregnancy reduces the presence of iron deficit and maternal ferropenic anemia, compared with no supplementation in healthy women and without anemia. This RCT has some limitations, such as the fact that it is not double blind, consists of a small sample and there has been a large number of abandonments. The CG found in the performed research, regarding the recommendations on iron supplementation in pregnancy, are not unanimous ( Table 2 ).

**Table 2:** Clinical Guidelines included

Reference	Recommendations	SR
U.S. Preventive Services Task Force (2015) 2	Evidence on the effect of universal iron supplementation on asymptomatic pregnant women on maternal clinical parameters is insufficient. More evidence is needed, and the balance between benefits and risks cannot be determined.	**C**
Direção-Geral da Saúde (2013) 9	Supplementation with iron should be individualized, based on clinical-laboratory parameters such as blood count, ferritin and CRP in the first consultation and at 28 weeks. Make the determination of serum ferritin before starting iron, and only prescribe if ferritin < 70 ng/mL.	**C**
Direção-Geral da Saúde (2015) 8	Start supplementation with 30–60 mg/day of elementary iron between 14 and 16 weeks and 6 days, in the absence of contraindications.	**C**
National Institute for Health and Care Excellence (2019) 16	Iron supplementation should not be offered in a universal way to all pregnant women, since it has no benefits on maternal health and may have unpleasant side effects.	**C**
Areia et al. 17 (2019)	Perform universal screening of anemia in pregnancy, with blood count and ferritin in the preconception and/or 1 ^st^ trimester, between 24 and 28 weeks of pregnancy, and in the 3 ^rd^ trimester. In women without anemia, supplementation should be initiated in pregnant women with ferritin < 30 ng/mL - daily oral administration of at least 60 mg elemental iron (consider intermittent administration on nonconsecutive days to decrease side effects and increase absorption and adherence).	**B**
Pavord et al. (2011) 7	Iron deficiency anemia in pregnancy is common and associated with increased risk of maternal morbidity and mortality. Routine screening with serum ferritin, outside the context of research, to diagnose anemia in pregnancy is not currently recommended. Individual approach, based on the results of anemia screening, as well as identification of women at increased risk. Nonanemic women at risk of iron deficiency should be identified and either started on prophylactic iron empirically (40–80 mg of elemental iron once a day) or have serum ferritin checked first (and iron offered if ferritin < 30 ug/l).	**C**

Abbreviations: CRP, C-reactive protein; SR, strength of recommendation.

The U.S. Preventive Services Task Force (USPSTF), 2 in its CG published in 2015 ( Table 2 ), states that there is consistent evidence of increased maternal levels of Hb and ferritin, establishing that the evidence is inconsistent regarding whether this increase leads to improved maternal clinical outcomes in nonanemic pregnant women. The USPSTF concludes that the evidence on the effect of universal iron supplementation on asymptomatic pregnant women on maternal outcomes is insufficient and that more evidence is needed for the balance between benefits and risks to be determined.

The CG of the DGS “Approach, diagnosis and treatment of ferropenia in adult” published in 2013 ( Table 2 ), refers to the early diagnosis of ferropenia in the prenatal period, as it reduces transfusion needs. 9 To this end, it suggests that serum ferritin should be determined before starting iron, and that it should only be prescribed if ferritin < 70ng/mL. They also state that iron supplementation should be individualized, based on clinical-laboratory parameters such as blood count, ferritin, and C-reactive protein (CRP) at the 1 ^st^ consultation and at 28 weeks.

The CG of the DGS – National Low-Risk Pregnancy Surveillance Program – published in 2015 ( Table 2 ) was elaborated with the main objective of defining a set of recommendations and appropriate interventions in the preconception, pregnancy and puerperium. 8 Regarding the supplementation with oral iron concerns, it determines that supplementation with between 30 and 60 mg/day of elemental iron should be started between 14 and 16 weeks and 6 days, in the absence of contraindications.

The National Institute for Health and Care Excellence (NICE) 16 presents a CG published initially in 2008, but updated in February 2019 ( Table 2 ), which aims to provide information on best practices for the clinical follow-up of healthy pregnant women with a single fetus low-risk pregnancy. Among other recommendations, it argues that iron supplementation should not be offered universally to all pregnant women, since if does not benefit maternal health and may have unpleasant side effects.

The Portuguese Society of Obstetrics and Maternal-Fetal Medicine (SPOMMF, in the Portuguese acronym) issued a CG in July 2019 ( Table 2 ) on the approach to anemia during pregnancy and postpartum, which explains that there is no consensus on the universal and systematic supplementation with iron in pregnant women to improve maternal and neonatal outcomes. 17 It also says that recent studies even indicate that unnecessary iron supplementation is associated with an increased risk of adverse outcomes such as preterm birth, low birthweight, and gestational diabetes. In this way, it recommends the universal screening of anemia in pregnancy, with blood count and ferritin in the preconception and/or 1 ^st^ trimester, between 24 ^th^ and 28 ^th^ weeks of pregnancy, and in the 3 ^rd^ trimester of pregnancy. For iron supplementation, in women without anemia, supplementation with ferritin < 30 ng/mL should be initiated in pregnant women by daily oral administration of at least 60 mg elemental iron. It also considers intermittent administration, on nonconsecutive days, to decrease side effects and increase absorption and adherence.

The British Committee for Standards in Hematology ( Table 2 ) issued a recommendation in 2011 to address iron deficiency in pregnancy as a strategy to prevent ferropenia. At the date of the article review process for submission, the authors verified that there is an update to this guideline published in October 2019, so it was decided to include the updated version, whose purpose was to provide health professionals with recommendations on prevention, diagnosis, and treatment of iron deficiency in pregnancy and in the postpartum period. 7 They state that iron deficiency anemia in pregnancy is common and is associated with increased risk of maternal morbidity and mortality. Regarding the diagnosis of anemia in pregnancy, they report that the optimal strategy is unknown, but routine screening with serum ferritin outside the context of research is not currently recommended. They present a list of indications for empirical iron supplementation and/or serum ferritin evaluation: anemic women in whom testing serum ferritin is necessary prior to iron supplementation (known hemoglobinopathy, before the parenteral replacement of iron); nonanemic women with high risk of iron depletion for empirical iron treatment with/without serum ferritin testing (previous anemia, 3 or more deliveries, twin or higher order multiple pregnancy, consecutive pregnancy < 1 year after delivery, vegetarians/vegans, pregnant teenagers, recent history of clinically significant bleeding); nonanemic women in whom serum ferritin may be necessary (high risk of bleeding during pregnancy or at birth, women declining blood products, such as Jehovah's Witnesses, women for whom providing compatible blood is challenging).

Although it was proposed by some authors, routine screening using serum ferritin is not recommended due to the costs, delays, and limitations of the parameter, and a detailed clinical history is preferred to identify which pregnant women meet the criteria. A serum ferritin level < 30 ug/l in pregnancy is indicative of iron deficiency, but higher levels do not rule out iron deficiency or depletion. In short, as a general recommendation, the authors argue that nonanemic women at risk of iron deficiency should be identified and either started on prophylactic iron empirically (between 40 and 80 mg of elemental iron once a day) or have serum ferritin checked first (and iron offered if ferritin < 30ug/l). Thus, they conclude that there is insufficient evidence to assess the benefits and potential hazards of routine iron supplementation for all women in pregnancy.

## Discussion

Prophylactic iron supplementation leads to an improvement in serum iron and hematological parameters in pregnancy. However, its correlation with maternal health benefits and risks is not well documented, with inconsistent results.

Of the scientific articles included in our analysis, the fact that they are directed to the disease stands out, having no proper correlation between the prevention of maternal anemia and the health benefits and quality of life for pregnant women.

Regarding the CG of the DGS existing in Portugal, for which most family doctors are governed, we consider that they need updating. The guidelines of the National Program for Low Risk Pregnancy Surveillance are based on the publication of the “Guideline: Daily Iron and Folic Acid supplementation in Pregnant Women” of the WHO, from 2012, 18 which in turn is based on exclusively laboratory maternal parameters, namely, reduction of the risk of maternal anemia at term and iron deficit at term. 8 18 Regarding clinical results, only the prevention of low weight at birth is mentioned, but this is not the objective of our review. A note also for the CG on the diagnosis and treatment of ferropenia in adults that, in our opinion, presents inconsistent data. This CG considers a ferritin value of 70 ng/ml as indicative for prescription of oral iron, using as bibliographic reference the British Committee for Standards in Haematology (2012 version), where the serum ferritin value taken as the lower limit for starting oral supplementation is 30 ng/ml and not 70 ng/ml. 9 19 Even in the updated version of the guideline on which they are based, the cutoff value of ferritin for supplementation remains 30 ng/ml.

We consider that there are some limitations in this review, mainly due to the heterogeneity between the included studies and MA, justified by the variable sample size, the different inclusion criteria, the variability of the daily elemental iron dose and iron type and the variability of micronutrients or of other vitamins associated with iron in the intervention groups, which leads to inconsistent results. High-quality RCTs are required, with more homogeneous methodologies, involving a greater number of pregnant women, to obtain more robust conclusions on the efficacy and safety of iron supplementation in all pregnant women for the prevention of maternal anemia. It is necessary to correlate the improvement of laboratory parameters in pregnant women without previous anemia with the possible improvement of clinical parameters. The great variability in the prevalence of anemia in pregnancy described in the introduction of this article may justify the difficulty in reaching consensus on the recommendations on universal supplementation, constituting itself a limitation to the present review.

To complement the present review, it would also be useful to evaluate the evidence on the impact of universal prophylactic iron supplementation on fetal outcomes and delivery.

## Conclusion

Given the different results between Tables 1 e 2 , it is possible to conclude that prophylactic iron supplementation in pregnancy is controversial and should not be performed universally in asymptomatic pregnant women (SORT C).
